# Current cancer burden in China: epidemiology, etiology, and prevention

**DOI:** 10.20892/j.issn.2095-3941.2022.0231

**Published:** 2022-08-30

**Authors:** Maomao Cao, He Li, Dianqin Sun, Siyi He, Xinxin Yan, Fan Yang, Shaoli Zhang, Changfa Xia, Lin Lei, Ji Peng, Wanqing Chen

**Affiliations:** 1 Office of Cancer Screening, National Cancer Center/National Clinical Research Center for Cancer/Cancer Hospital, Chinese Academy of Medical Sciences and Peking Union Medical College/Chinese Academy of Medical Sciences Key Laboratory for National Cancer Big Data Analysis and Implement, Beijing 100021, China; 2 Department of Cancer Prevention and Control, Shenzhen Center for Chronic Disease Control, Shenzhen 518020, China

**Keywords:** Cancer burden, risk factor, prevention, China

## Abstract

Cancer has become the most common cause of death in China. Owing to rapid economic development, improved livelihood, and shifts in risk factors, cancer epidemiology has experienced substantial changes during the past several decades. In this review, we aim to describe the current cancer epidemiology of the main types of cancer in China, report major risk factors associated with cancer development, and summarize the contributions of the Chinese government to controlling the cancer burden. A total of 4,064,000 new cases were diagnosed in China in 2016. The most frequent types are lung cancer (828,100; 20.4%), colorectal cancer (408,000; 10.0%), and gastric cancer (396,500; 9.8%). Lung (657,000; 27.2%), liver (336,400, 13.9%), and stomach (288,500; 12.0%) cancers are the 3 most deadly cancers in the general population. The 5-year survival rate for cancer has dramatically increased in recent decades. However, liver and particularly pancreatic cancers still have the poorest prognosis. The main modifiable risk factors associated with cancer development include infectious agents, smoking, alcohol consumption, obesity, unhealthful dietary habits, and inadequate physical activity. The Chinese government has made unremitting efforts to decrease the cancer burden, including cancer education and investment in cancer screening programs.

## Introduction

Cancer, with its chronic development and tendency to be fatal, has become one of the leading causes of death in China^1^. Over the past several decades, China has experienced groundbreaking economic development, transitioning from an agrarian economy to an economic superpower. The standard of living and health resources have dramatically improved, and lifestyles have become increasingly urbanized and westernized. In addition, the multiple levels of government in China have continually engaged in cancer prevention and control efforts, such as implementing educational programs and screening for harmful cancers. The patterns of cancer epidemiology have changed dramatically.

Cancer epidemiology information is critical for providing effective and timely guidance in establishing healthcare policies and intervention measures. Therefore, cancer epidemiology data require timely updating. In this review, we aim to describe the current trends in cancer epidemiology, report the relevant risk factors for various cancers, and highlight China’s efforts to ease the burden of cancer in recent decades.

To provide convincing evidence, we extracted relevant data from published articles in several electronic databases (e.g., PubMed, China National Knowledge Infrastructure, and Google Scholar), books, and official websites, such as the People’s Republic of China, the National Bureau of Statistics, National Health Commission of the People’s Republic of China, and the World Health Organization (WHO). Search terms included, but were not limited to, “cancer epidemiology”, “cancer burden”, “incidence”, “mortality”, “contribution”, “cancer prevention”, and “risk factors”.

## Cancer epidemiology

### Incidence trends

**Figure 1:**
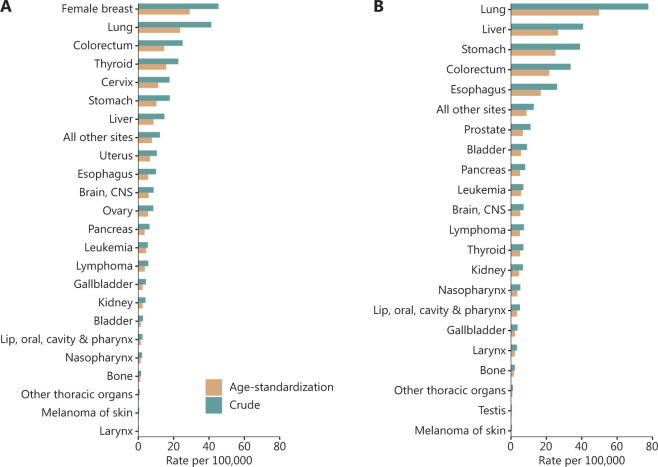
Crude and age-standardized incidence of cancer by sex (A. female, B. male). Data were extracted from Cancer incidence and mortality in China, 2016^1^.

According to the estimates of the National Cancer Center (NCC) of China^1^, approximately 4,064,000 newly diagnosed cancer cases were reported in 2016, thus resulting in a crude incidence rate of 293.9/100,000. The age-standardized incidence rate (ASIR) in the world standard population for all cancer cases combined was 186.5/100,000. Men had a higher crude incidence rate or ASIR than women for most types of cancer (**Figure 1**). The most frequently diagnosed cancer types were those of the lung, colorectum, stomach, liver, and breast, accounting for 20.4%, 10.0%, 9.8%, 9.6%, and 7.5%, respectively, and representing 57.3% of all cancers combined (**Table 1**). The most common cancer in men was lung cancer (549,800; 24.6%), which was followed by liver cancer (288,800; 12.9%), stomach cancer (276,300; 12.4%), colorectal cancer (238,500; 10.7%), and esophageal cancer (184,500; 8.3%). In contrast, the most frequent malignancy in women was female breast cancer (306,000; 16.7%), and lung cancer (278,300; 15.2%) was the second most prevalent, and was followed by colorectal cancer (169,500; 9.3%) and thyroid cancer (152,600; 8.3%)^1^. The ratio of frequency is >1 for most cancers in men and women (excluding sex-specific malignancies, e.g., cancers of the prostate and female breast), except for cancers of the gallbladder, brain, and thyroid (0.9, 0.9, and 0.3, respectively). The highest ratio is for laryngeal cancer (10.7).

**Table 1:** The most frequent cancers in China by sex, 2016

Site	Overall			Men			Women		
	Cases	Percentage (%)	Rank	Cases	Percentage (%)	Rank	Cases	Percentage (%)	Rank
All sites	4,064,000	100.0	–	2,234,300	100.0	–	1,829,600	100.0	–
Lung	828,100	20.4	1	549,800	24.6	1	278,300	15.2	2
Colon-rectum	408,000	10.0	2	238,500	10.7	4	169,500	9.3	3
Stomach	396,500	9.8	3	276,300	12.4	3	120,200	6.6	5
Liver	388,800	9.6	4	288,800	12.9	2	100,000	5.5	7
Female breast	306,000	7.5	5	–	–	–	306,000	16.7	1
Esophagus	252,500	6.2	6	184,500	8.3	5	68,000	3.7	10
Thyroid	202,600	5.0	7	50,000	2.2	12	152,600	8.3	4
All other sites	173,600	4.3	8	90,900	4.1	6	82,700	4.5	8
Cervix	119,300	2.9	9	–	–	–	119,300	6.5	6
Brain, CNS	109,000	2.7	10	50,500	2.3	11	58,500	3.2	11
Pancreas	100,400	2.5	11	57,000	2.6	9	43,400	2.4	13
Lymphoma	89,900	2.2	12	51,600	2.3	10	38,300	2.1	14
Leukemia	85,800	2.1	13	49,400	2.2	13	36,400	2.0	15
Bladder	82,300	2.0	14	64,200	2.9	8	18,000	1.0	18
Prostate	78,300	1.9	15	78,300	3.5	7	–	–	–
Kidney	75,800	1.9	16	48,000	2.1	14	27,800	1.5	17
Uterus	71,100	1.7	17	–	–	–	71,100	3.9	9
Ovary	57,200	1.4	18	–	–	–	57,200	3.1	12
Gallbladder	55,700	1.4	19	26,400	1.2	17	29,300	1.6	16
Lip, oral, cavity, and pharynx	52,200	1.3	20	36,200	1.6	16	16,100	0.9	19
Nasopharynx	52,000	1.3	21	37,400	1.7	15	14,700	0.8	20
Bone	25,800	0.6	22	14,900	0.7	19	10,900	0.6	21
Larynx	25,700	0.6	23	23,500	1.1	18	2,200	0.1	24
Other thoracic organs	13,100	0.3	24	7,600	0.3	20	5,500	0.3	22
Melanoma of skin	7,000	0.2	25	3,500	0.2	21	3,500	0.2	23
Testis	3,400	0.1	26	3,400	0.2	22	–	–

Cancer incidence and mortality in China, 2016^1^.

The crude incidence rates for all cancers gradually increased in men and women from 2000 to 2015. However, the total cancer incidence rate in both sexes combined significantly decreased after age standardization^2^. Specifically, for both sexes, a downward trend in ASIR was seen for digestive tract cancer, including esophageal cancer, stomach cancer, and liver cancer. Lung cancer, colorectal cancer, stomach cancer, breast cancer, and thyroid cancer remained the most common cancers in China^1,3^.

Individually, the ASIR in men showed a stable trend over the past several decades. A substantial increase in prostate cancer incidence was observed, with an average annual percentage change of 7.1% from 2000 to 2016, particularly between 2000 and 2005 (annual percentage change of 12.5%)^1^. The incidence rates of leukemia and cancers of the colorectum, brain, pancreas, and bladder showed upward trends. Cancer incidence after age standardization was on a rise by 2.3% per year in women^1^. Trends for cancers of the esophagus, stomach, and liver steadily decreased during the same period. However, the ASIRs for colorectal cancer, uterine cancer, cervical cancer, and lung cancer have increased generally. Notably, the incidence of thyroid cancer rapidly increased, and its average annual percentage change was estimated to be 20.6% from 2007 to 2016^1^.

### Mortality trends

**Figure 2:**
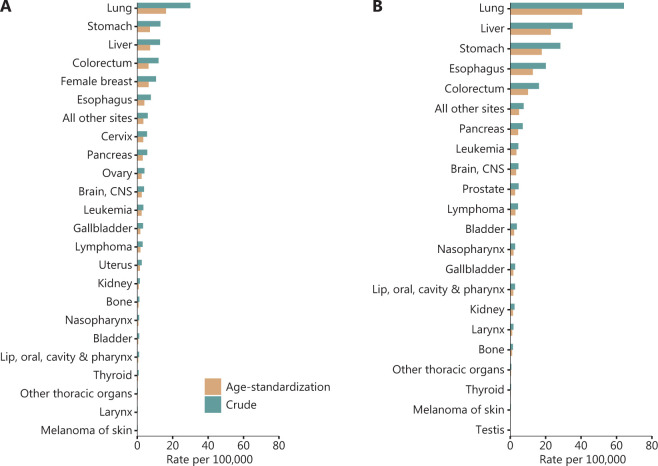
Crude and age-standardized mortality of cancer by sex (A. female, B. male). Data were extracted from Cancer incidence and mortality in China, 2016^1^.

The cancer mortality ranking estimated by the NCC is shown in **Table 2**. In 2016, 2,413,500 cancer deaths were recorded in China, with a crude mortality rate of 174.6/100,000. The age-standardized mortality rate (ASMR) in the world standard population for all cancers combined was 105.2/100,000. Men had a significantly higher ASMR than women (138.1/100,000 *vs.* 74.0/100,000)^1^. The mortality rate by sex is shown in **Figure 2**. The highest numbers of cancer deaths were those for lung cancer (657,000; 27.2%), with a crude mortality rate of 47.5/100,000, liver cancer (336,400; 13.9%), stomach cancer (288,500; 12.0%), colorectal cancer (195,600; 8.1%), and esophageal cancer (193,900; 8.0%), representing 69.2% of all cancer deaths. Approximately 29.7% of all deaths from cancer were ascribed to lung cancer in men. The second most common cancer in men was liver cancer (249,600; 16.3%), which was followed by stomach cancer (200,200; 13.1%), esophageal cancer (142,300, 9.3%), and colorectal cancer (114,500; 7.5%). The leading causes of cancer death in women were lung cancer (202,300; 22.9%), stomach cancer (88,400; 10.0%), liver cancer (86,800; 9.8%), colorectal cancer (81,000; 9.2%), and breast cancer (71,700; 8.1%). The ratio of mortality of frequency for men to women was <1 for gallbladder cancer (0.9) and thyroid cancer (0.6), and the highest ratio was found for laryngeal cancer (7.4).

**Table 2:** Leading causes of death in China by sex, 2016

Site	Overall			Men			Women		
	Deaths	Percentage (%)	Rank	Deaths	Percentage (%)	Rank	Deaths	Percentage (%)	Rank
All sites	2,413,500	100.0	–	1,530,700	100.0	–	882,800	100.0	–
Lung	657,000	27.2	1	454,700	29.7	1	202,300	22.9	1
Liver	336,400	13.9	2	249,600	16.3	2	86,800	9.8	3
Stomach	288,500	12.0	3	200,200	13.1	3	88,400	10.0	2
Colon–rectum	195,600	8.1	4	114,500	7.5	5	81,000	9.2	4
Esophagus	193,900	8.0	5	142,300	9.3	4	51,600	5.8	6
All other sites	93,400	3.9	6	53,500	3.5	6	39,900	4.5	7
Pancreas	87,900	3.6	7	49,800	3.3	7	38,100	4.3	8
Female breast	71,700	3.0	8	–	–	–	71,700	8.1	5
Brain, CNS	58,500	2.4	9	32,600	2.1	9	25,900	2.9	11
Leukemia	55,700	2.3	10	32,400	2.1	10	23,200	2.6	12
Lymphoma	51,500	2.1	11	31,000	2.0	11	20,500	2.3	14
Gallbladder	41,400	1.7	12	19,500	1.3	14	22,000	2.5	13
Cervix	37,200	1.5	13	–	–	–	37,200	4.2	9
Bladder	33,700	1.4	14	26,200	1.7	12	7,500	0.9	18
Prostate	33,600	1.4	15	33,600	2.2	8	–	–	–
Ovary	27,200	1.1	16	–	–	–	27,200	3.1	10
Kidney	26,900	1.1	17	17,100	1.1	16	9,800	1.1	16
Nasopharynx	26,700	1.1	18	19,700	1.3	13	7,000	0.8	19
Lip, oral, cavity, and pharynx	25,800	1.1	19	18,900	1.2	15	6,900	0.8	20
Bone	18,400	0.8	20	10,700	0.7	18	7,700	0.9	17
Uterus	17,100	0.7	21	–	–	–	17,100	1.9	15
Larynx	14,300	0.6	22	12,500	0.8	17	1,700	0.2	23
Thyroid	8,300	0.3	23	3,100	0.2	20	5,200	0.6	21
Other thoracic organs	6,800	0.3	24	4,300	0.3	19	2,500	0.3	22
Melanoma of skin	3800	0.2	25	2,100	0.1	21	1,700	0.2	24
Testis	900	0.0	26	900	0.1	22	–	–	–

Cancer incidence and mortality in China, 2016^1^.

According to estimates from the National Bureau of Health Statistics, the 4 leading causes of death in China from 2000 to 2020 were cancer, heart conditions, cerebrovascular disease, and respiratory disease (**Table 3**). In urban areas, cancer remains the major cause of death, whereas in rural regions, it has overtaken respiratory diseases and become the leading cause of death^4^. Furthermore, the mortality rank for major cancer types has changed according to the 3 nationwide retrospective sampling surveys of causes of death^5^. **Table 4** lists the 10 most common cancers in China over the past 3 periods (1973–1975, 1990–1992, and 2004–2005). In the first and second National Retrospective Sampling Survey of Death Causes, stomach cancer was the most deadly cancer (accounting for 23.0% and 23.2% of all cancer deaths, respectively). However, in the third National Retrospective Sampling Survey of Death Causes, lung cancer became the most common cause of death (accounting for 22.7% of all cancer deaths). The 5 leading causes of cancer-associated death have gradually become lung cancer, liver cancer, stomach cancer, esophageal cancer, and colorectal cancer^6^.

**Table 3:** Leading causes of death in China

Year	Rural (%)				Urban (%)			
	Cancer	Heart conditions	Cerebrovascular disease	Respiratory disease	Cancer	Heart conditions	Cerebrovascular disease	Respiratory disease
2000	18.4	12.4	18.4	23.1	24.4	17.7	21.3	13.3
2001	17.7	13.1	19.0	22.5	24.9	17.6	20.4	13.4
2002	20.7	14.3	17.3	15.6	23.5	14.6	17.5	15.6
2003	25.3	12.0	23.8	18.7	25.5	14.4	20.0	14.6
2004	23.7	12.5	14.9	13.3	23.9	18.8	19.1	13.1
2005	20.3	11.8	21.2	23.5	22.9	17.9	21.2	12.6
2006	25.1	13.9	20.4	16.4	27.3	17.1	17.7	13.1
2007	24.8	14.8	20.6	17.2	28.5	16.3	18.0	13.1
2008	25.4	14.1	21.7	16.9	27.1	19.7	19.6	11.9
2009	24.3	17.2	23.2	15.0	27.0	20.8	20.4	10.5
2010	23.1	17.9	23.4	14.2	26.3	20.9	20.2	11.0
2011	23.6	19.4	21.7	13.3	27.8	21.3	20.2	10.6
2012	23.0	18.1	20.6	15.8	26.8	21.5	19.6	12.3
2013	22.4	21.9	22.9	11.5	25.5	21.6	20.3	12.4
2014	23.0	21.7	22.9	12.1	26.2	22.1	20.4	12.0
2015	23.2	21.8	23.2	12.1	26.4	22.0	20.6	11.8
2016	22.9	22.2	23.3	12.0	26.1	22.6	20.6	11.2
2017	23.1	22.7	23.2	11.6	26.1	23.0	20.6	10.9
2018	23.0	23.5	23.2	11.2	26.0	23.3	20.5	10.8
2019	23.3	23.8	22.9	10.8	25.7	23.7	20.6	10.4
2020	23.1	24.5	23.5	9.1	25.4	24.6	21.3	8.7

National Bureau of Statistics of China.

**Table 4:** Major causes of cancer-associated death from 1973 to 2005

1973–1975 (year)		1990–1992 (year)		2004–2005 (year)	
Site	Proportion (%)	Site	Proportion (%)	Site	Proportion (%)
Stomach	23.0	Stomach	23.2	Lung	22.7
Esophagus	22.3	Liver	18.8	Liver	19.3
Liver	15.1	Lung	16.2	Stomach	18.2
Cervix	7.6	Esophagus	16.1	Esophagus	11.2
Lung	7.4	Colorectum	4.9	Colorectum	5.2
Colorectum	5.3	Leukemia	3.4	Leukemia	2.8
Leukemia	3.8	Cervix	1.8	Brain	2.3
Nasopharynx	2.8	Nasopharynx	1.6	Breast	2.1
Breast	2.0	Breast	1.6	Pancreas	1.9
Brain	1.9	Bladder	0.9	Bone	1.3
Others	8.8	Others	11.5	Others	13.0

Three National Retrospective Sampling Survey of Death Causes^6^.

Regarding the ASMR in men, a steadily descending trend has been observed for all cancer types combined, with an annual percentage change of 1.2%^1^. The mortality decreased significantly for cancers of the esophagus, stomach, and liver. Additionally, upward trends in mortality were observed for prostate cancer, pancreatic cancer, brain cancer, and leukemia. In women, the ASMR for esophageal, stomach, and liver cancers also decreased dramatically during this period, whereas the rates for cervical and thyroid cancers showed increasing trends. The trend in the ASMR for cancers of the colorectum, brain, and uterus remained stable^1,3^.

### Survival trends

**Figure 3:**
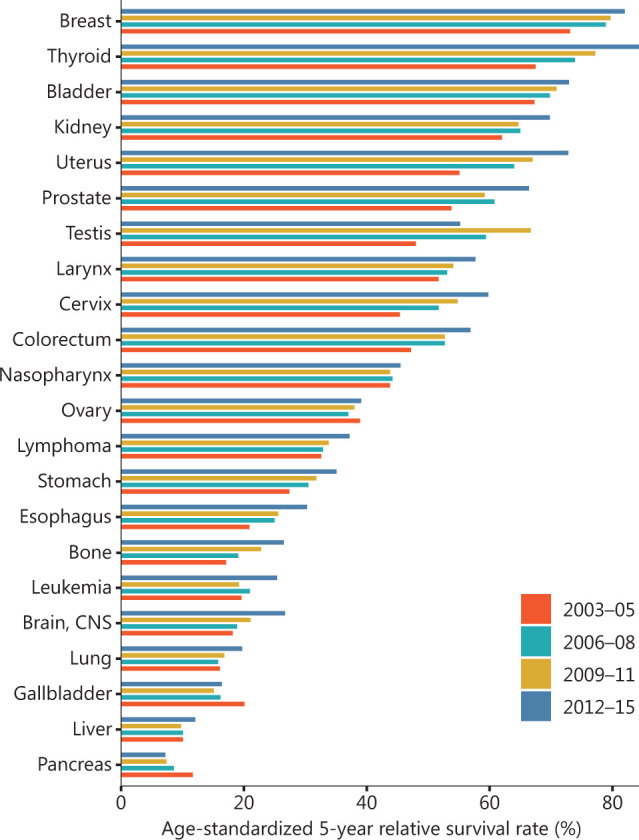
Age-standardized 5-year relative survival rates for the common cancers. Data were extracted from Changing cancer survival in China during 2003–2015: a pooled analysis of 17 population-based cancer registries^7^.

**Figure 3** illustrates the prognosis for the major types of cancer. The age-standardized 5-year survival rate was estimated to be 40.5% in 2012–2015, representing a substantial increase with respect to 2003–2005 (30.9%)^7^. Women had better prognosis than men (47.8% *vs.* 33.9%). The age-standardized 5-year survival rate for overall cancers combined in urban areas was higher than that in rural areas (46.7% *vs*. 33.6%)^7^.

The age-standardized 5-year survival rate substantially varied for individual cancers. The best prognosis across the 4 periods was observed for thyroid cancer (67.5% in 2003–2005, 73.9% in 2006–2008, 77.2% in 2009–2011, and 84.3% in 2012–2015). Breast cancer had the second highest 5-year survival rate (82.0%) and was followed by bladder cancer (72.9%) in 2012–2015. The lowest 5-year age-standardized survival rate (7.2%) was found for pancreatic cancer (**Figure 3**)^7^. In urban areas, the 5-year age-standardized survival rate increased from 39.5% in 2003–2005 to 46.7% in 2012–2015, with a 2.2% average change. In contrast, greater growth in the 5-year standardized survival rate was observed in rural than urban areas: the rate increased from 21.8% in 2003–2005 to 33.6% in 2012–2015, with a 3.9% average change^7^.

## Etiology and underlying modifiable risk factors

### Infectious agents

Infectious agents generally include viruses, bacteria, and parasites, which can result in cancer or increase cancer risk. The most common infectious causes associated with cancer development are hepatitis B/C virus (HBV/HCV), aflatoxin exposure, *Helicobacter pylori* (*H. pylori*), human papillomavirus (HPV), and Epstein-Barr virus, which are risk factors for liver cancer, gastric cancer, cervical cancer, and nasopharyngeal cancer, respectively.

HBV and HCV are the predominant established risk factors for liver cancer in China. Approximately 53.2% of liver cancer deaths in China have been attributed to HBV infection, and 17.0% of deaths have been attributed to HCV infection^8^. The main infectious risk factors for liver cancer also include exposure to dietary aflatoxins. The population attributable risk of aflatoxin-associated liver cancer has been estimated to be 25% in China^9^. *H. pylori* has been classified as a class I carcinogen by the WHO^10^. A case-control study has shown that 78.5% of non-cardia gastric cancer and 62.1% of gastric cardia cancer cases are attributable to *H. pylori* infection^11^. The overall prevalence rate of *H. pylori* reached 55% among Chinese residents^12^. Although a slowly downward trend in the prevalence rate of *H. pylori* in China has been observed, the decline has not been sufficient to affect the incidence of gastric cancer^13^. Of the more than 100 identified HPV types, HPV-16 and HPV-18 were categorized as human carcinogens in 1995 by the International Agency for Research on Cancer^14^. The national overall prevalence of HPV infection showed a slightly decreasing trend from 16.4% in 2003 to 17.3% in 2016^15^, and approximately 97.4% of deaths caused by cervical cancer in China are attributable to HPV infection^16^. In addition, several epidemiological studies have suggested that HPV infection is associated with the development of not only cervical cancer but also other types of cancer, including head and neck squamous cell carcinoma, oral, pharyngeal, and laryngeal cancers, and skin cancer^17–19^.

In general, infectious factors play major roles in the development and progression of various cancers, and approximately one-third of cancer cases in China are attributable to infection. In 2018, 780,000 of the 2.2 million new infection-attributable cases worldwide were in China^20^.

### Alcohol

Alcohol consumption is considered a primary risk factor for cancer mortality worldwide. Alcohol consumption has been classified as a human carcinogen, and is known to generate reactive oxygen species, impair the body’s ability to break down nutrients, and increase the estrogen levels in the blood^21^. According to WHO estimates, 4.4% and 1.0% of deaths from cancer in men and women, respectively, are attributable to alcohol consumption in China^22^. A meta-analysis of 26 observational studies has indicated that alcohol consumption is significantly associated with increased risk of liver cancer, esophageal squamous cell carcinoma, and stomach cancer^23^. Alcohol consumption can also increase the risk of cancer in a dose-responsive manner^24^. Even at extremely low levels of consumption, alcohol has been found to increase the risk of head-and-neck cancers and esophageal squamous cell carcinoma^25^. A systematic analysis from the Global Burden of Disease Study has suggested that the optimal level of alcohol consumption for minimizing harm is zero^26^, thus suggesting that no safe level of alcohol consumption exists. The number of liters of alcohol consumed by the Chinese population 15 years of age or above has stably increased from 1960 to 2015^22^. The prevalence rates of heavy episodic drinking in men and women (older than 15 years) are 36.3% and 8.6%, respectively, significantly higher than those in other countries^22^. Thus, the interventions and policies for the control of alcohol consumption should be reinforced.

### Tobacco

A substantial number of studies have shown that tobacco use is associated with increased risk of many cancer types, such as lung cancer, liver cancer, stomach cancer, and esophageal cancer^27–29^. Tobacco products can produce multiple carcinogens, such as nicotine and volatile organic compounds, which cause complex mutations in critical cancer genes^30^. A population-based cohort study conducted in Austria has indicated that current smokers, compared with people who have never smoked, have greater risk of cancers of the lung (hazard ratio [HR] = 17.66, 95% CI, 14.65–21.29), larynx (HR = 11.29, 95% CI, 5.49–23.20), head and neck (HR = 2.53, 95% CI, 1.87–3.41), esophagus (HR = 3.84, 95% CI, 2.33–6.35), liver (HR = 4.07, 95% CI, 2.55–6.51), bladder (HR = 3.08, 95% CI, 2.00–4.73), pancreas (HR = 2.68, 95% CI, 1.93–3.71), and colorectum (HR = 1.31, 95% CI, 1.09–1.57)^28^. A nationwide prospective study in China recruiting 0.5 million adults has suggested that smoking significantly elevates the risk of lung cancer (risk ratio [RR] = 2.51, 95% CI, 2.18–2.90), liver cancer (RR = 1.32, 95% CI, 1.12–1.54), stomach cancer (RR = 1.34, 95% CI, 1.16–1.55), and esophageal cancer (RR = 1.47, 95% CI, 1.24–1.73)^31^. Furthermore, a positive dose-response relationship with smoking intensity and duration has been observed for most cancers^32–34^. Second-hand smoke, including environmental exposure to tobacco smoke, is also classified as a known human carcinogen by a series of regulatory agencies and governmental authorities^35^. Studies have indicated that passive smoking can increase the risk of cancer among people who do not smoke^36–39^.

China is the largest producer and consumer of tobacco worldwide. More than 300 million smokers live in China, and over 700 million non-smokers are exposed to second-hand smoke at least once per day each week^40^. Despite the initiation of tobacco control policies and educational activities, only minor effects have been observed on the smoking prevalence in China. Six nation-wide epidemiological surveys of smoking status, conducted in 1984, 1996, 2002, 2010, 2015, and 2018, have shown that the prevalence rate of smoking remains high, although a slight decrease was observed from 33.9% to 26.6%^41^. Data from the National Health Service Surveys have also indicated that the implementation of tobacco control policies in China has not been effective in decreasing smoking prevalence: the proportion of current smokers was 26.0% in 2003, 24.9% in 2008, and 25.2% in 2013^42^. Thus, actions to strengthen the tobacco surveillance system are urgently needed.

### Diet

Many epidemiological studies have reported the association between cancer outcomes and dietary factors^43–45^. A Mediterranean diet, associated with longevity and high quality of life, has been shown to significantly decrease the risk of cancer incidence and mortality^46^. Vegetable consumption has been identified to have a protective role against cancer. Compared with non-vegetarians, vegetarians have a statistically significantly lower overall cancer risk^44^. Moreover, vegan diets have been found to decrease overall cancer incidence by 16% in both sexes and to decrease female-specific cancer incidence by 34%^47^. Raw garlic consumption has been inversely associated with the development of liver cancer among individuals negative for HBV^48^. The Women’s Health Initiative Randomized Trial has indicated that a low-fat dietary pattern, characterized by high vegetable, fruit, and grain intake, can decrease the risk of death from breast cancer in postmenopausal women^43^. However, a prospective cohort study has suggested no association between dietary fat and liver cancer risk^49^.

Moreover, some methods of preserving and cooking food can influence cancer risk^50^. People who prefer salty food, and salt-preserved meat and fish, generally have a higher risk of gastric cancer^51^. A recent meta-analysis of 11 case-control and cohort studies has indicated that a high intake of salt increases the risk of gastric cancer dramatically (odds ratio = 2.05, 95% CI, 1.60-2.62)^52^. Consumption of processed meat may increase the risk of breast cancer^45^. Red meat intake has been found to increase the risk of overall cancer by 31% and the risk of breast cancer by 83%^53^. Dietary exposure to aflatoxin, derived mainly from grain mildew, is the primary etiologic factor influencing liver cancer in China. A widespread change in dietary habits from consumption of maize to rice, and changes in storage methods, have effectively decreased aflatoxin contamination in food in China, thus decreasing liver cancer burden^50,54^. Chinese-style salted fish has been classified as a carcinogen by the International Agency for Research on Cancer^55^. However, a recent population-based case-control study in Southern China has shown no association between consumption of hard Chinese-style salted fish and nasopharyngeal carcinoma^56^. Dietary habits in different regions of China are diverse, and thus the evidence of other dietary factors contributing to cancer development is currently insufficient^57^.

### Physical activity

Strong evidence indicates that regular physical activity, particularly at high intensity, may negatively affect the risk of several cancers^58^. Physical activity decreases the risk of cancer by lowering levels of sex hormones, decreasing insulin sensitivity, and increasing colonic peristalsis^59^. Individuals with the highest level of recreational or occupational physical activity, compared with those with the lowest level, have a 15% lower risk of bladder cancer, according to a systematic literature review and meta-analysis of 15 studies^60^. A prospective cohort study in 73,049 Chinese women has also suggested that exercise at or above the recommended level is associated with a 27% lower risk of breast cancer than lower levels of exercise^61^. A negative statistical association between ovarian cancer risk and physical activity has been reported in a study conducted in Southern China^62^. A case-control study based on data from eastern and southern China has also reported that physical activity is beneficial in decreasing the risk of prostate cancer^63^. In comparison, a sedentary lifestyle has emerged as a risk factor for several site-specific cancers. Physical inactivity is responsible for 8.9% of the colorectal cancer burden in men and 9.0% of that in women^64^. A positive statistical association between ovarian cancer risk and sitting time has been reported in a quantitative meta-analysis^65^. China had a high prevalence of insufficient activity in adolescents in 2016 (84.3%), indicating a slight decrease from 85.4% in 2001^66^. Boys have been found to have higher activity than girls (prevalence rates of insufficient activity of 80.1% and 89.1%, respectively)^66^. Policy actions to improve physical activity should be prioritized, particularly policies targeted to girls.

### Overweight and obesity

Overweight and obesity can cause long-lasting inflammation and oxidative stress^67^. According to estimates, 11.9% and 13.1% of the cancer burdens in men and women, respectively, are attributable to obesity^68^. Robust evidence has shown that obesity increases the risk of endometrial cancer, esophageal adenocarcinoma, and cancers of the colorectum, postmenopausal breast, prostate, and kidney^69^. A prospective cohort of 68,253 Chinese women has indicated that obesity increases the risk of overall cancer (HR = 1.36, 95% CI = 1.21–1.52), postmenopausal breast cancer (HR = 2.43, 95% CI = 1.73–3.40), endometrial cancer (HR = 5.34, 95% CI = 3.48–8.18), liver cancer (HR = 1.93, 95% CI = 1.14–3.27), and epithelial ovarian cancer (HR = 2.44, 95% CI = 1.37–4.35)^70^. Moreover, a meta-analysis of more than 10 million participants from 24 prospective studies has found a positive association between high body mass index (BMI) and gastric cardia cancer^71^. A BMI-dependent increase in the risk of developing liver cancer has been reported in a systematic review of 28 prospective studies^72^. In addition, obesity leads to a high risk of development of type 2 diabetes and thus increases the risk of colorectal cancer^73^.

Obesity has become a major public health problem in China, with prevalence values of 34.3% for overweight (BMI: 24.0–27.9 kg/m^2^) and 16.4% for obesity (BMI: 28.0 kg/m^2^ or higher) in adults (≥18 years), according to the Chronic Disease and Nutrition Surveillance 2015–2019 survey^74^. By 2030, the prevalence rate of overweight and obesity is expected to reach 65.3% in adults, equivalent to 790 million people^75^. The high rates of obesity and overweight are driven by a combination of factors including economic growth, socio-cultural norms, and substantial changes in dietary patterns^74^. National prevention and control policies toward obesity have been insufficient, and thus this issue requires more government attention^75^.

## Cancer prevention and control

**Figure 4:**
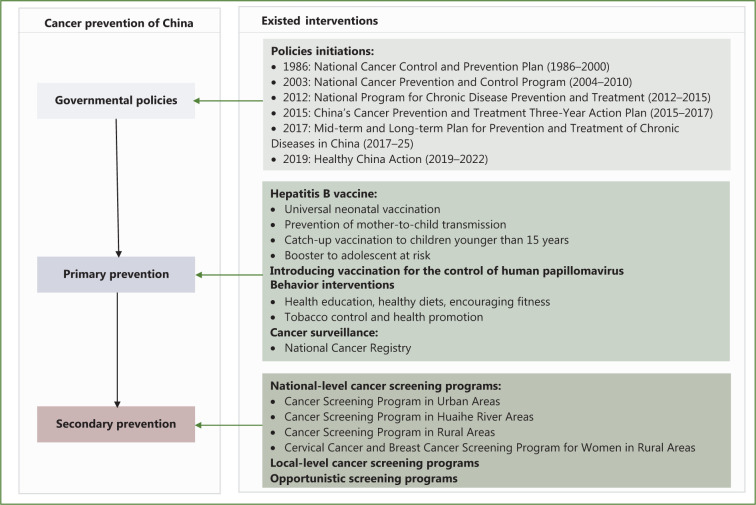
Measures for cancer prevention and control in China.

To control the cancer burden, the Chinese government has made substantial efforts over the past decades. A series of policies were initiated in different periods according to social development and requirements. The main strategies for decreasing cancer burden have focused mainly on primary and secondary prevention, as shown in **Figure 4**.

### Governmental guidance

To guide cancer prevention in an orderly and effective manner, China has issued the “National Cancer Prevention and Control Plan (1986–2000)”, the first programmatic documentation of cancer prevention and control in China^76^. Cancer prevention has been strongly emphasized. The release of the National Cancer Prevention and Control Program (2004–2010) by the Chinese Ministry of Health endeavored to establish a national cancer registry system, enhance health education at the population level, and expand screening coverage for the principal cancers, particularly in rural areas observing obvious achievement in narrowing the urban-rural gap of the worse cancer incidence^77^. To address increases in non-communicable diseases and advance public health development, the National Program for Chronic Disease Prevention and Treatment (2012–2015)^78^ and China’s Cancer Prevention and Treatment Three-Year Action Plan (2015–2017)^79^ were initiated collaboratively by the Chinese government. Expanding the coverage of services for timely detection and treatment of frequently occurring cancers, emphasis is placed on the management of high-risk populations and the promotion of healthful lifestyles (e.g., tobacco cessation) in these 2 documents.

On October 25, 2016, the Chinese government enacted the “Healthy China 2030” strategy, jointly issued and implemented by the Central Committee of the Communist Party of China and the State Council, drafting a blueprint for cancer control^80^. In 2019, the Healthy China Action Plan (2019–2022) was officially implemented in accordance with the “Healthy China 2030” strategy^81^. The guidelines aim to improve cancer screening by increasing screening coverage and the early detection rates of major cancers in high-incidence areas. In addition, the promotion of healthful eating, physical activity, and other health initiatives is emphasized. The policies and programmatic documents issued in past decades have provided effective guidance and indications for cancer prevention in various regions, thus reflecting the government’s attention to cancer prevention and control.

### Primary prevention

Primary prevention efforts are considered the most cost-effective strategy to curb the cancer burden. These efforts are aimed at decreasing exposure to risk factors associated with cancer development and increasing the resistance of the population to addressing cancer risk—a high priority aspect in cancer control. Among all efforts, vaccination and educational interventions targeting risk factors are the major preventive measures.

#### Vaccination

Several vaccines are available targeting several specific cancers caused by viruses. Hepatitis B vaccine has high efficacy in preventing the development of liver cancer. This vaccine is administered to newborns at 0 months of age (or within 24 h of birth), 1 month, and 6 months^82,83^. To increase hepatitis B vaccine coverage, the central government of China initiated several programmatic documents and policies, such as waiving vaccine administration and purchase costs^84^. In 2002, the hepatitis B vaccine was incorporated into the Expanded Program of Immunization, and a Global Alliance for Vaccines and Immunization was also implemented to ensure the vaccine’s availability in China’s poorest provinces and counties^85^. Encouragingly, the coverage rate of 3 doses of the hepatitis B vaccine has markedly increased, from 70.7% in 1999 to 99.7% in 2012^86^. According to the national serological surveys conducted in 1992 and 2006, the infection rate of HBV has significantly decreased from 9.8% to 7.2%^84^, thus suggesting that the vaccination program for the control of the transmission of HBV in China has made great achievements.

The HPV vaccine is expected to decrease cervical cancer incidence and thus eliminate cervical cancer^87^. The WHO Cervical Cancer Elimination Modelling Consortium has reported that elimination could occur between 2059 and 2102 when girls vaccinated only in all countries^88^. Currently, the first Chinese HPV vaccine, Cecolin, has gained prequalification from WHO^89^. However, the HPV vaccine coverage in China remains unsatisfactory. A survey conducted at Peking University in China involving 884 college students has indicated that only 9.5% of the female students had received the vaccine^90^. Many misunderstandings regarding HPV and HPV vaccines persist among the public, thus suggesting that limited knowledge regarding the HPV vaccine is the primary barrier to acceptance of the vaccine^91^. Beyond limited awareness, high price may be another important reason limiting vaccination coverage. Generally, HPV vaccines cost as much as $271–450 (RMB: 1806–2999) for the 3 doses; this high cost makes the HPV vaccine inaccessible for most people, particularly women living in rural areas^92^. To increase HPV vaccine coverage, health promotion campaigns associated with the HPV vaccine are required. Public media also should take responsibility for disseminating knowledge regarding the HPV vaccine in a simple manner to help more people to learn about its importance.

#### Behavioral interventions

Behavioral interventions refer to the provision of behavioral education and correcting actions that can increase the risk of cancer. The main health initiatives in China include health education, promoting healthful diets, fitness, and tobacco control. The NCC, the Chinese Center for Disease Control and Prevention, the Chinese Anti-Cancer Association, and other institutions or organizations produce several themed publicity events every year across the entire country^93^, to publicize essential information for cancer prevention and control, and enhance public awareness and acceptance of cancer prevention. These organizations also provide scientific guidance to the public for developing healthful diets through media or books.

At present, China’s tobacco control strategy is focused mainly on adolescents, passive smokers (particularly women and children), and current smokers. To address tobacco use, the following measures have been implemented according to the Ottawa Charter: (1) enacting of public policies, such as banning the sale of cigarettes to those under 18 years of age; (2) elimination of exposure to second-hand tobacco smoke in all workplaces, public places, family environments, and public transportation; and (3) implementation of standardized tobacco packaging with large graphic or text warnings about the harms of smoking/tobacco use to health on all packages^94^. To limit the transmission of *H. pylori*, a comprehensive national prevention strategy had been implemented in China, including promoting good hygiene and handwashing, and the use of serving chopsticks and spoons^95^. Salt is an essential component of the human diet, however, high intake can significantly increase the risk of stomach cancer^96^. The WHO has called for all countries to decrease their intake of salt to less than 5 g/day by the year 2025^97^. The Healthy Lifestyle Campaign for All, initiated by the Disease Control Bureau of the Ministry of Health in 2007^98^, focused on a balanced diet and physical activity. A series of salt control and educational initiatives were subsequently launched. The results of Chronic Disease and Nutrition Surveillance performed in 31 provinces (autonomous regions and municipalities) from 2015 to 2019 has described the achievements of salt reduction initiatives in 2020: the per capita daily consumption was 9.3 grams of salt, 1.2 grams lower than that in 2015^99^. These accomplishments indicate improved health awareness among the public.

### Cancer registry

Cancer epidemiology, which includes cancer incidence, morbidity, mortality, and survival, is crucial to provide scientific information for cancer prevention and control. As the basis of the cancer control programs, the cancer registry plays a crucial role in cancer epidemic surveillance, evaluation of cancer control programs, and guidance for anti-cancer strategies. In China, cancer surveillance registration emerged in the 1960s in 3 demonstration sites: Qidong (population-based causes of death registry in 1958), Linxian (esophageal cancer incidence registry in 1959), and Shanghai (population-based cancer registry in 1963)^100^. The system was designed primarily to collect data on cancer incidence, mortality, and survival, as well as the population distribution in each region, and to further describe the burden of cancer. The cancer registration system developed very slowly at the beginning of its establishment. To enhance the management of the surveillance system, the Reporting Manual of Cancer Registration was released in 1982 by the Office of National Central Cancer Registry. In 2002, the National Central Cancer Registry was launched under the supervision of the former Ministry of Health, and was responsible for systematically collecting, analyzing, and interpreting cancer data. In 2008, to improve the standard of public health service, the cancer registration program was incorporated into the Central Subsidies for Local Transfer Payments by the Ministry of Finance combined with the former Ministry of Health. With strong support from the Chinese government, the national cancer surveillance network has rapidly developed. By 2009, 149 cancer registries had been established, accounting for 10% of the national population^100^. Every year, the NCC estimates the numbers of new cancer cases and deaths in China according to the collected data, thus providing key evidence for the formulation of policies. By the end of 2020, cancer registration covered 1152 counties, accounting for approximately 40% of the national population (598 million)^1^.

### Cancer screening

The risk factors associated with cancer development are diverse, and thus decreasing cancer burden through only primary prevention is difficult. A substantial number of high-quality epidemiological studies have shown that screening for certain types of cancer may effectively decrease cancer incidence/mortality through enabling early detection and lesion removal in asymptomatic at-risk populations^101–103^. Four large-scale population-based organized cancer screening programs in mainland China have been initiated since 2005: Early Detection and Early Treatment of Cancer in Rural Cancer (2005), Early Detection and Early Treatment of Cancer in Huaihe Areas (2007), Cervical Cancer and Breast Cancer Screening Program for Women in Rural Areas (also called Two Cancer Screening; 2009), and Cancer Screening Program in Urban China (2012), to provide free screening services for people at high risk and those who are living in high-risk areas. The screening includes the 8 most common cancers in China: those of the lung, stomach, liver, colorectum, esophagus, cervix, breast, and nasopharynx^104^. The Chinese government has greatly expanded the coverage of cancer screening programs. From 2009 to 2018, the coverage of Two Cancer Screening expanded from 200 counties (cities, districts) to more than 1,700 counties, providing free cervical cancer screening for 85 million women and breast cancer screening for 20 million women^105^. Esophageal cancer, gastric cancer, and liver cancer were all incorporated into 3 national screening programs. As of 2018, 3.7 million high-risk individuals were identified, and a total of 0.2 million individuals had received free screening for upper gastrointestinal cancer in rural high-risk areas, thus yielding an early detection rate of 75.1% and a treatment rate of 85.7%^106^.

Beyond the programs supported by the central government, regional screening programs have also been implemented in several areas. In 2017, “Combined Screening for Common Cancers” was initiated in the Tianjin Municipal area, thus providing screening for lung cancer, breast cancer, gastric cancer, and liver cancer. The joint screening program for common cancers has been piloted in 48 communities and towns in 6 districts and Jizhou districts, and more than 150,000 residents have received cancer screening services^107^. Colorectal cancer screening at no cost is offered to residents in Zhejiang Province every 5 years^108^. Beyond the organized cancer screening programs, opportunistic screening may serve as an alternative, given the increasing health awareness of the public, the high accessibility of medical services, and improvements in service quality. Since 2019, the number of demonstration sites for opportunistic screening have increased. By the end of 2020, 997 units in rural areas have provided opportunistic screening services for upper gastrointestinal cancer^109^.

## Expenditures and national insurance for cancer control

In recent years, the central government of China has paid great attention to the implementation of cancer screening, and funding for cancer screening has rapidly grown in recent decades. Since 2005, 280 million RMB each year has been invested by the central government to support the screening programs in the rural high-risk, Huaihe, and urban areas^106^. In addition, China’s government has invested more than 600 million RMB to screen for breast cancer and cervical cancer, and 19 provinces (municipalities and districts) achieved full coverage of screening for these 2 cancer types in 2020 through combined funding from multiple local governments^110^.

High expenditure and financial burden are important obstacles to the accessibility of cancer medical treatment. The Chinese government has launched 6 rounds of national drug price negotiation since 2016 to lower the costs for innovative drugs, particularly for anticancer drugs^111^. Recent price negotiation with pharmaceutical companies for medications covered by national basic health insurance was conducted in December 2021 by China’s National Healthcare Security Administration. Among the 117 drugs under negotiation, 74 were successfully added to the 2021 National Reimbursement Drug List, thus resulting in a 61.7% price decrease^112^. Moreover, 18 of the 74 newly added drugs were anti-cancer medicines, thereby greatly alleviating the personal financial burden of cancer treatment among Chinese residents. Beyond increasing insurance coverage for anti-cancer drugs, basic reimbursement services for examinations, treatment, and other costs for major diseases have steadily increased^113^. With the help of national subsidies, disadvantaged residents have gained access to convenient health services.

## Limitations and challenges in cancer prevention

Geographical differences in diverse modifiable cancer risk factors have resulted in cancer burdens that vary across regions in China, thus leading to substantial difficulties in primary cancer prevention. Health educational campaigns in different regions should be improved and directed well. In addition, the HPV vaccine has not been covered in national immunization in China, thus limiting the number of vaccinations. Therefore, a larger budget should be allocated to cancer prevention in the future.

In general, because cancer occurrence is influenced by many risk factors, screening among the general population is not cost-effective. Risk-adapted screening strategies have been used for most current cancer screening in China^114,115^. By considering the epidemiological characteristics of the population and related biological markers or genetic factors, the risk prediction model provides an effective method for focusing coverage on high-risk individuals precisely. With the growth of big data and biological information, more accurate biomarkers have been identified, such as circulating tumor DNA^116^. Therefore, the established risk prediction models should be updated in the future. Additionally, how to choose accurate risk prediction models and set suitable cut-off values for the risk scores used in screening will be future research priorities to improve screening efficiency.

In conclusion, current epidemiological data indicate that cancer remains the major factor threatening health in China. Clear incremental trends in the incidence of thyroid and breast cancer in women, and colorectal and prostate cancer in men, have been observed. The great improvements in living conditions, particularly in rural areas of China, progress in health awareness, and shifts in risk factors are thought to be the main reasons for the dramatic changes in cancer burden observed in the past several decades. Hence, the central government and multidisciplinary institutions require strategies to strengthen existing measures in cancer prevention, screening, diagnosis, and management.
